# Allosteric Cholesterol Site in Glycine Receptors Characterized through Molecular Simulations

**DOI:** 10.1021/acs.jpcb.4c01703

**Published:** 2024-05-15

**Authors:** Farzaneh Jalalypour, Rebecca J. Howard, Erik Lindahl

**Affiliations:** † Science for Life Laboratory, Department of Applied Physics, KTH Royal Institute of Technology, 17121 Solna, Sweden; ‡ Science for Life Laboratory, Department of Biochemistry and Biophysics, Stockholm University, 17121 Solna, Sweden

## Abstract

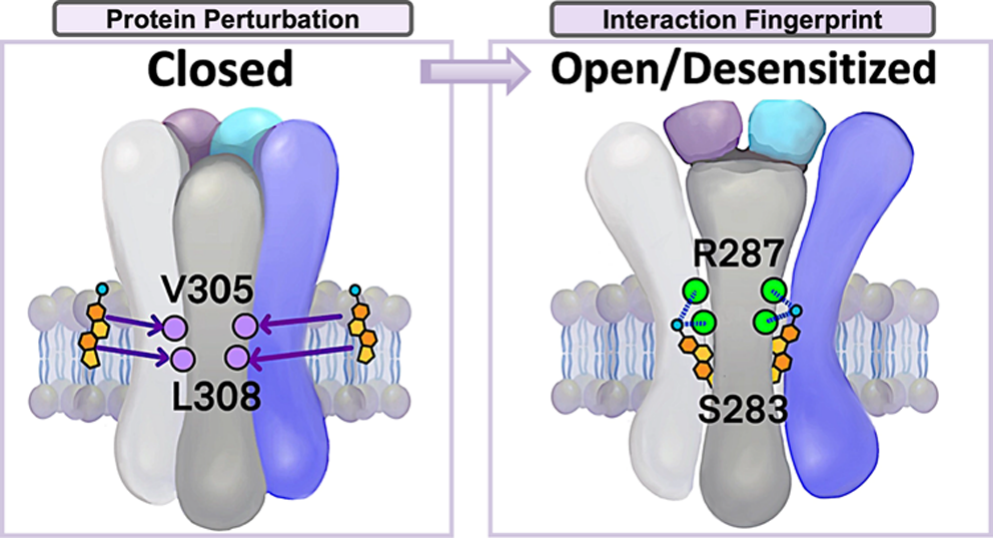

Glycine receptors are pentameric ligand-gated ion channels that conduct chloride ions across postsynaptic membranes to facilitate fast inhibitory neurotransmission. In addition to gating by the glycine agonist, interactions with lipids and other compounds in the surrounding membrane environment modulate their function, but molecular details of these interactions remain unclear, in particular, for cholesterol. Here, we report coarse-grained simulations in a model neuronal membrane for three zebrafish glycine receptor structures representing apparent resting, open, and desensitized states. We then converted the systems to all-atom models to examine detailed lipid interactions. Cholesterol bound to the receptor at an outer-leaflet intersubunit site, with a preference for the open and desensitized versus resting states, indicating that it can bias receptor function. Finally, we used short atomistic simulations and iterative amino acid perturbations to identify residues that may mediate allosteric gating transitions. Frequent cholesterol contacts in atomistic simulations clustered with residues identified by perturbation analysis and overlapped with mutations influencing channel function and pathology. Cholesterol binding at this site was also observed in a recently reported pig heteromeric glycine receptor. These results indicate state-dependent lipid interactions relevant to allosteric transitions of glycine receptors, including specific amino acid contacts applicable to biophysical modeling and pharmaceutical design.

## Introduction

Pentameric ligand-gated ion channels (pLGICs) mediate cellular communications by converting chemical signals to electrical signals. The classical example involves a presynaptic neuron releasing a neurotransmitter such as acetylcholine or γ-aminobutyric acid, which binds to a pLGIC on the postsynaptic neuron. This agonist interaction induces an allosteric conformational change in the receptor, which leads to pore opening, ion conduction, and membrane potential alteration.^1^ Apart from their primary agonists, pLGICs are sensitive to a range of allosteric modulators, small molecules that can either amplify or reduce the effect of the main agonist.^2−5^ This property has enabled recreational drugs throughout the history of mankind, and it is the basis of several modern clinical drugs including anesthetics.^6−8^ However, the precise mechanisms underlying the pLGIC modulation remain unclear.

Members of the pLGIC family can be divided into two main groups: cationic channels, such as the nicotinic acetylcholine receptor (nAChR), which play primarily excitatory roles in neurotransmission, and anionic channels, such as the type-A γ-aminobutyric acid-A receptor (GABA_A_R),^1^ which feature heavily in neuro-inhibition. Both groups exhibit conserved structural and functional features, including an extracellular domain (ECD) responsible for agonist binding, a transmembrane domain (TMD) forming the central pore for ion conductance, and an intracellular domain (ICD) of a variable sequence. Each TMD subunit contains four helices (M1–M4) connected by extracellular and intracellular loops, and five such subunits create the complete TMD. In the resting state, the channel is closed via a hydrophobic constriction near the middle of the membrane plane. Agonist binding in the ECD triggers a series of conformational changes, including expansion of the hydrophobic gate nearly 50 Å from the neurotransmitter sites, which eventually leads to pore opening. In most eukaryotic pLGICs, this transient open state undergoes a rapid, subtle transition to a desensitized state with the ligand still bound but where the pore is occluded at an alternative gate facing the cytoplasm. Both gating and allosteric modulation involve ligand binding and conformational changes between and within subunits.^9,10^

Recent structures of the glycine receptor (GlyR), an anionic pLGIC, provide notable insights into the architecture and functional cycling of these channels. The GlyR is a major inhibitory chloride channel in the spinal cord and brain and it is the target of several allosteric modulators.^2,3^ Functional GlyRs include both homomeric assemblies of α1, α2, or α3 subunits^11,12^ and heteromeric combinations of four α-subunits and one β-subunit.^13−15^ One recent study reported structures of the homomeric zebrafish α1 GlyR in multiple apparent functional states, including conformations annotated as closed, open, closed, desensitized, and “super-open,” processed from the same cryo-EM dataset in the presence of the partial agonist taurine.^11^ These receptors were extracted directly from insect cell membranes using the styrene maleic acid (SMA) copolymer, enabling the preservation of embedding lipids from the expression conditions, although in this case, no specific lipids were resolved in the final structures. Shortly thereafter, a structure of the native pig α1β GlyR was reported in an apparent desensitized state with the agonist glycine.^13^ Subsequent agonist-bound structures of heteromeric GlyRs from the zebrafish and pig largely recapitulated this asymmetric desensitized state.^14,15^ These structures provide opportunities to investigate state-dependent lipid interactions and allostery in both homo- and heteromeric systems.

Lipid interactions have received increasing attention in structure–function studies of membrane proteins. First, general physical characteristics of the lipid bilayer, such as fluidity, thickness, and curvature, have been shown to influence membrane protein properties.^16,17^ For instance, membrane thinning by rhomboid proteins has been shown to modify elastic properties required for rhodopsin function.^18^ Further, specific lipid types have been shown to bind selectively to transmembrane protein surfaces and modulate function. For example, specific phospholipids allosterically modulate G-protein-coupled receptors (GPCRs),^19^ and polyunsaturated fatty acids have been shown to modulate pLGICs^20,21^ and to shift activation of voltage-gated ion channels to less polarized potentials.^22^ Notably, the steroid lipid cholesterol stabilizes the structural elements of the β2 adrenergic receptor, a prototypical GPCR, and hinders sampling of its conformational landscape.^23^ The sodium–potassium ATPase pump is also more active when cholesterol is present. Function of the nAChR is similarly dependent on the lipid content as well as bulk fluidity, particularly on the presence of cholesterol.^24−26^ For LGICs, cholesterol has been shown to enhance agonist-induced channel opening as well as desensitization of the cationic nAChR, while its depletion reduces function.^26,27^ Cholesterol has also been proposed to bind specific sites in anionic pLGICs,^26,28,29^ but it has thus far been challenging to retain bound lipids during reconstitution of pLGICs for structure determination of complexes.

As an alternative approach, modern simulation methods may offer a range of insights into the complex dynamics of the membrane–protein system such as the GlyR. For instance, it has been shown that lipid localization and interactions with proteins can be predicted by long MD simulations.^16,30^ While coarse-grained simulations in particular remove much of the detailed interactions by grouping several atoms as beads to reduce the degrees of freedom,^31,32^ this approximation enables them to cover significantly longer time scales while retaining at least some qualitative accuracy. This can provide excellent sampling in complex systems limited by diffusion, and the results of coarse-grained simulations can then be converted back to all-atom models and used to initiate atomistic simulations to provide more detailed models of interactions, e.g., with lipids in specific sites in a dynamic protein. Coarse-grained simulations starting from homology models of homopentameric human GlyRs have recently been used to indicate that cholesterol preferentially binds to the open over the closed state.^29^

Here, we took advantage of recent GlyR structures in closed, open, and desensitized states determined under identical, nanodisc-embedded conditions to study computational cholesterol–protein dynamics.^11^ We leveraged coarse-grained and atomistic MD simulations to identify specific sites occupied by cholesterol in particular functional states across the conformational cycle. We also investigated cholesterol interactions in a heteromeric GlyR, and we related our findings to previous functional data, particularly in synthetic and disease variants. We also applied a modified perturbation response scanning approach^33,34^ to identify specific residues likely to mediate cholesterol effects on gating. Our results identified state-dependent lipid interactions capable of influencing allosteric transitions of the GlyR and amino acid contacts that could be targets for pharmaceutical design.

## Methods

### Coarse-Grained Starting Models

For simulations of homomeric GlyRs, starting models were taken from structures of SMA-extracted zebrafish α1 GlyRs in the presence of taurine.^11^ The selected models were classified from the same cryo-EM dataset as closed (PDB ID 6PM3), open (PDB ID 6PM2), and desensitized (PDB ID 6PM1)^11^ (Table 1). Simulations of the heteromeric GlyR were based on the pig α1β GlyR, whose amino acid sequence is 99% identical to the human GlyR, in the presence of glycine, solubilized from the native spinal cord in n-dodecyl-β-maltoside, and classified by cryo-EM as desensitized (PDB ID 7MLY).^13^ Since the ICD was not resolved in any of the experimental structures, it was replaced in our models by a short, flexible loop (Ala-328, Gly-329, Thr-330)^35^ using MODELER.^32^

**Table 1:** Starting Models Used for Simulations in This Study

PDB ID	type	subunit(s)	structural state	organism	resolution (Å)
6PM1	homopentameric	α1	desensitized	*Danio rerio*	3.0
6PM2	homopentameric	α1	open	*Danio rerio*	3.0
6PM3	homopentameric	α1	closed	*Danio rerio*	3.0
7MLY	heteropentameric	1 β/4 α1	desensitized	*Sus scrofa*	2.7

### Equilibrium Coarse-Grained MD Simulations in an Asymmetrical Neuronal Membrane

**Figure 1:**
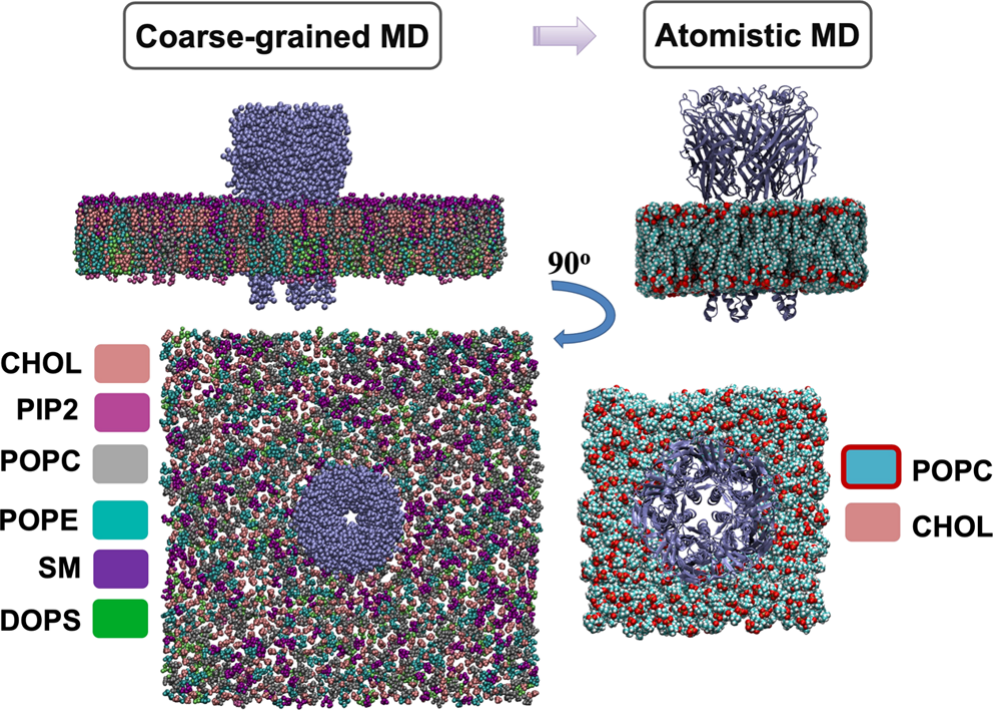
Simulation methods. Molecular simulations of GlyRs were carried out in three different functional states. Left-hand views are of a representative coarse-grained simulation box with multiatom beads representing the receptor (blue) and six different types of lipids (colored separately, according to the legend). Right-hand views illustrate an atomistic simulation of a back-mapped conformation, with the receptor represented as ribbons (blue) and cholesterol (pink) and POPC (cyan) represented as spheres, colored by heteroatom. Water and ions are hidden for the sake of clarity.

The MARTINI force field (Martini 2.2 amino acid, Martini 2.0 lipids, and nonpolarizable water)^32^ was employed for coarse-grained simulations (Figure 1), in which one backbone bead and 0–3 side chain beads represent each residue. Martini Bilayer Maker in CHARMM-GUI^36^ was used to insert each protein structure (closed, open, or desensitized) in an asymmetrical bilayer of lipids predicted in previous computational work to represent a neuronal membrane (Table 2).^29^ This mixture was based on multiple lipidomic analyses from the brain,^37^ where GlyRs populate regions including the olfactory bulb, cerebellum, and hippocampus.^38^ In animals including zebrafish and humans, GlyRs are even more highly expressed in spinal neurons than in the brain;^39^ although lipids in the spinal cord may modestly differ from the brain,^40−44^ they have yet to be computationally modeled in similar detail. The general neuronal distribution was therefore used to approximate the physiological environment for GlyRs. The outer leaflet contained 44.9% cholesterol (CHOL), 24.4% phosphatidylcholine (POPC), 11.2% phosphoethanolamine (POPE), and 19.5% sphingomyelin (SM), while the inner leaflet consisted of 43.3% CHOL, 13.4% POPC, 21.6% POPE, 3.1% SM, 16.5% dioleoylphosphatidylserine (DOPS), and 2.1% phosphatidylinositol 4,5-bisphosphate (PIP2). In all coarse-grained simulations, taurine ligands were removed. The systems were neutralized by the addition of ions to approximate 150 mM NaCl. In total, the box spanned 256 × 256 × 169 Å containing protein, lipids, ions, and nonpolarizable water. After energy minimization and equilibration, involving a gradual lowering of the force of the restraints, four replicas of each system were simulated for 22 μs each using GROMACS 2021,^45^ with CG scaffolds to restrain the protein secondary structure, while lipids, ions, and water were freely diffusing.

**Table 2:** Lipid Composition of the Asymmetrical Neuronal Membrane Mimic

lipid type	number in the outer leaflet (%)	number in the inner leaflet (%)
CHOL	528 (45%)	504 (43.3%)
POPC	288 (24.4%)	156 (13.4%)
POPE	132 (11.2%)	252 (21.6%)
DOPS	0 (0%)	192 (16.5%)
PIP2	0 (0%)	24 (2.1%)
SM	228 (19.4%)	36 (3.1%)
Total	1176 (100%)	1164 (100%)

### Occupancy Analysis and Binding Site Identification

Protein–lipid interactions were assessed by using two different approaches. First, the lipid occupancies during coarse-grained simulations were measured for each structural state. To this end, the distribution of each lipid type was estimated from the four 22 μs replicates and averaged over all frames using the Volmap tool in VMD.^46^ A grid with a resolution of 1 Å in each dimension was used to calculate the lipid occupancy. Lipid density was reported when the lipid and protein were in contact in a given position in at least 50% of the simulation frames. Second, PyLipID,^47^ a Python-based analysis tool, was used to further examine lipid sites including native-like binding poses and the interaction residence time of each residue.

### Atomistic Starting Models

To generate atomistic starting poses for each structure with cholesterol in its putative interaction site, we first back-mapped the final frame of an open-state coarse-grained simulation, including the protein and its five most closely associated cholesterol molecules, to atomistic representation in POPC using the CG2AA tool^48^ together with the bilayer builder in CHARMM-GUI.^36^ Following system equilibration, we then ran a preliminary 300 ns fully unrestrained simulation to relax cholesterol interactions at the protein–lipid interface. To obtain a reasonably symmetric starting state with similar binding in all sites of the homomeric channel, we then selected the cholesterol molecule with the lowest root-mean-square deviation (RMSD) from its initial pose as a best fit to the interfacial site and inserted it symmetrically at all five subunit interfaces of the closed, open, and desensitized starting models.

### Atomistic MD Simulations

To assess cholesterol binding, atomistic simulations of cholesterol-bound receptors in each state (prepared as described above) were simulated in quadruple replicates covering 300 ns each (Figure 1).^35^ Simulations were performed using GROMACS 2021^45^ with CHARMM36 force field parameters^49^ and the TIP3P water model. The systems were neutralized by adding ions to approximate 150 mM NaCl, and the simulation time step was set to 2 fs. The bilayer dimensions were 120 × 120 × 170 Å. LINCS^50^ was used to constrain the length of hydrogen bonds. The particle-mesh Ewald approach^51^ was used to estimate long-range electrostatic interactions. The Parrinello–Rahman barostat^52^ and v-rescale thermostat^53^ were used to maintain pressure (1 bar) and temperature (300 K), respectively. Pore characteristics in each state were analyzed using the channel annotation package (CHAP).^54^

### Atomistic Contact and Interaction Assessment

Cholesterol contacts with the receptor were first assessed by measuring the distance between the oxygen atom of the hydroxyl group of each cholesterol molecule and the hydrogen atom (HG1) of the residue Ser-283 using Python MDAnalysis scripts.^55^ Then, the Protein–Ligand Interaction Fingerprints (ProLIF 1.0.0) tool^56^ was used to generate an interaction map, screening all potential interactions using the default distance cutoffs of 3.5 Å for hydrogen bonds and 4.5 Å for hydrophobic, π–π, and cation−π interactions.

### Protein Perturbation Calculations

In order to investigate protein dynamics, a second set of simulations was initiated from the same atomistic starting structures but removing the glycine agonist and again performing quadruple replicate simulations for 300 ns in each state. The technique of protein perturbation, which has previously been used to investigate the conformational modulation of proteins,^33,34,57,58^ relies on a covariance matrix derived from MD simulations to relate external forces to shifts in atomic coordinates according to the principles of linear response theory. The coarse-grained representation of each state was constructed by considering each Cα atom as a node. Then, numerous random forces were sequentially applied in various directions to each node, generating displacement values. To attain a desired state, the objective is to identify a residue and the direction in which it needs to be perturbed to generate effective displacements. The anticipated displacements are compared to the difference between closed and open structures, which in turn is used to identify residues whose response to perturbations has high overlap with the gating conformational transition as candidate hotspots, where previous studies have indicated ∼0.6 as a threshold for significance.^33^

In this study, the protein RMSD and pore characteristics of GlyR models were measured to verify the stability of the structure during simulations (Figure S1). Parameters including the trajectory interval and number of perturbations were optimized based on previous protocols^33^ to maximize sampling and achieve converged overlap values. Each residue of the pentameric receptor was subsequently perturbed by 1000 different force vectors. Three separate calculations were performed to cover the closed-to-open, open-to-desensitized, and desensitized-to-closed conformational transitions. For each transition, an equilibrated structure was chosen from the frames within the simulation to serve as the starting point for the transition since the perturbation approach requires a minimized and relaxed conformation as described by the force field. The experimental structures were used as the target state. The perturbation approach applies random forces, and sampling was enhanced by repeating each perturbation calculation five times for each transition. Since the structural difference between the open and desensitized conformations is limited (mostly localized to a ∼1 Å contraction in the inner pore radius) and because this transition is not expected to involve communication between ECD and TMD, we do not expect this transition to indicate any significant overlap values, but we rather include it as a reference to check that the perturbation approach does not generate spurious high correlations.

## Results

### State-Dependent Site for Cholesterol Identified by Coarse-Grained Simulations

**Figure 2:**
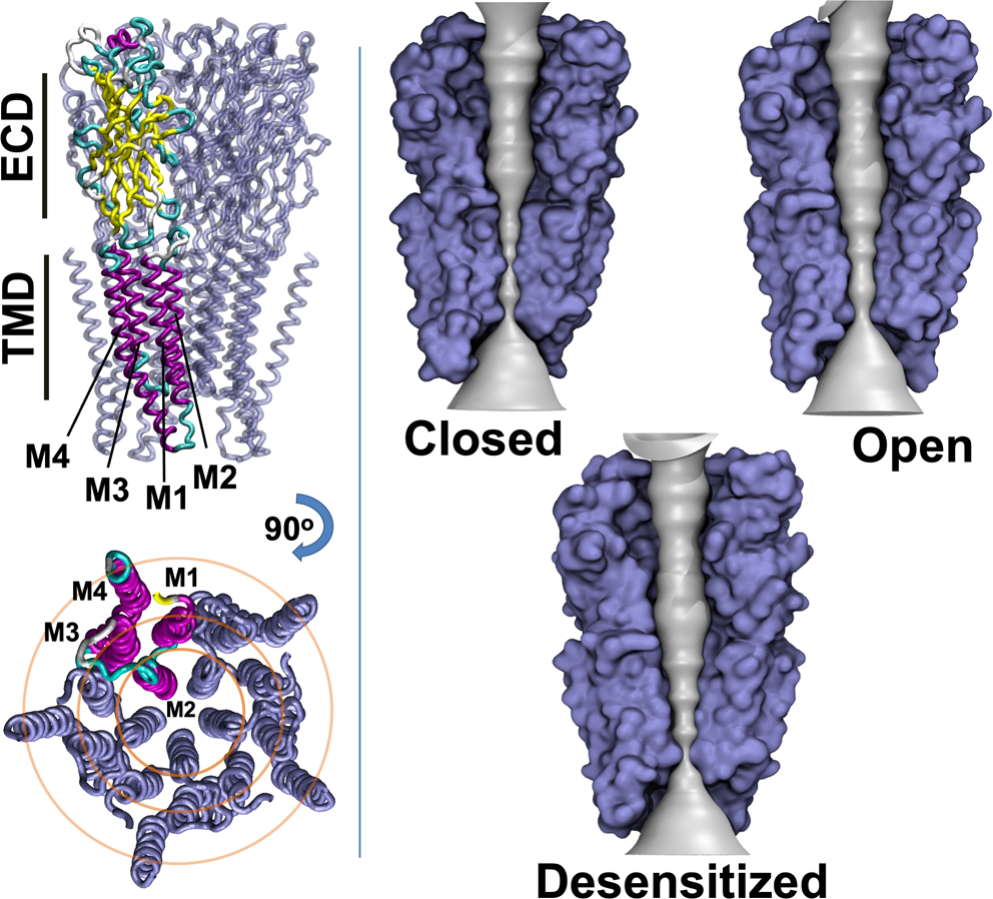
GlyR structural templates. Rightmost views show the schematics of a homomeric GlyR (purple), with one subunit colored by the secondary structure (yellow, β strands; magenta, α helices; cyan, loops) and transmembrane helices labeled (M1–M4). Due to a lack of structural information, the ICD is not shown. The upper view is from the membrane plane, and the lower view is of the TMD from the extracellular side. Left-hand views show GlyR cryo-EM structures determined in the presence of taurine in closed (PDB ID 6PM3), open (PDB ID 6PM2), and desensitized (PDB ID 6PM1) states. Structures are represented as surfaces (purple), with the first two subunits removed for clarity, revealing the linear pore (gray).

To characterize lipid interactions in all three functional states, we first applied coarse-grained MD simulations to cryo-EM structures of the lipid-embedded full-length zebrafish α1 GlyR (PDB IDs 6PM3, 6PM2, and 6PM1) determined at ≤3.2 Å resolution from three distinct classes of the same cryo-EM dataset.^11^ Although all the above-mentioned structures contained the partial agonist taurine in the extracellular ligand-binding site, the TMDs were notably distinct from one another, in particular, between resting and open states. The first was closed, similar to a resting state; the other two were comparable to open and desensitized structures determined in the presence of other partial or full agonists. In the context of lipid interactions, which occur primarily in the TMD, these three structures were therefore presumed to represent closed, open, and desensitized states. As introduced by Barrantes and colleagues,^59^ the five sets of GlyR M1–M4 helices can be conceived as three concentric rings (Figure 2). The central ring consists of the M2 helices surrounding the ion pore. An intermediate ring consists of the M1 and M3 helices, while the M4 helices constitute an outermost ring, largely embedded in the surrounding membrane. Lipids interact extensively with the M4 helices, to a lesser extent with peripheral residues of M1 and M3, and only rarely with M2.

**Figure 3:**
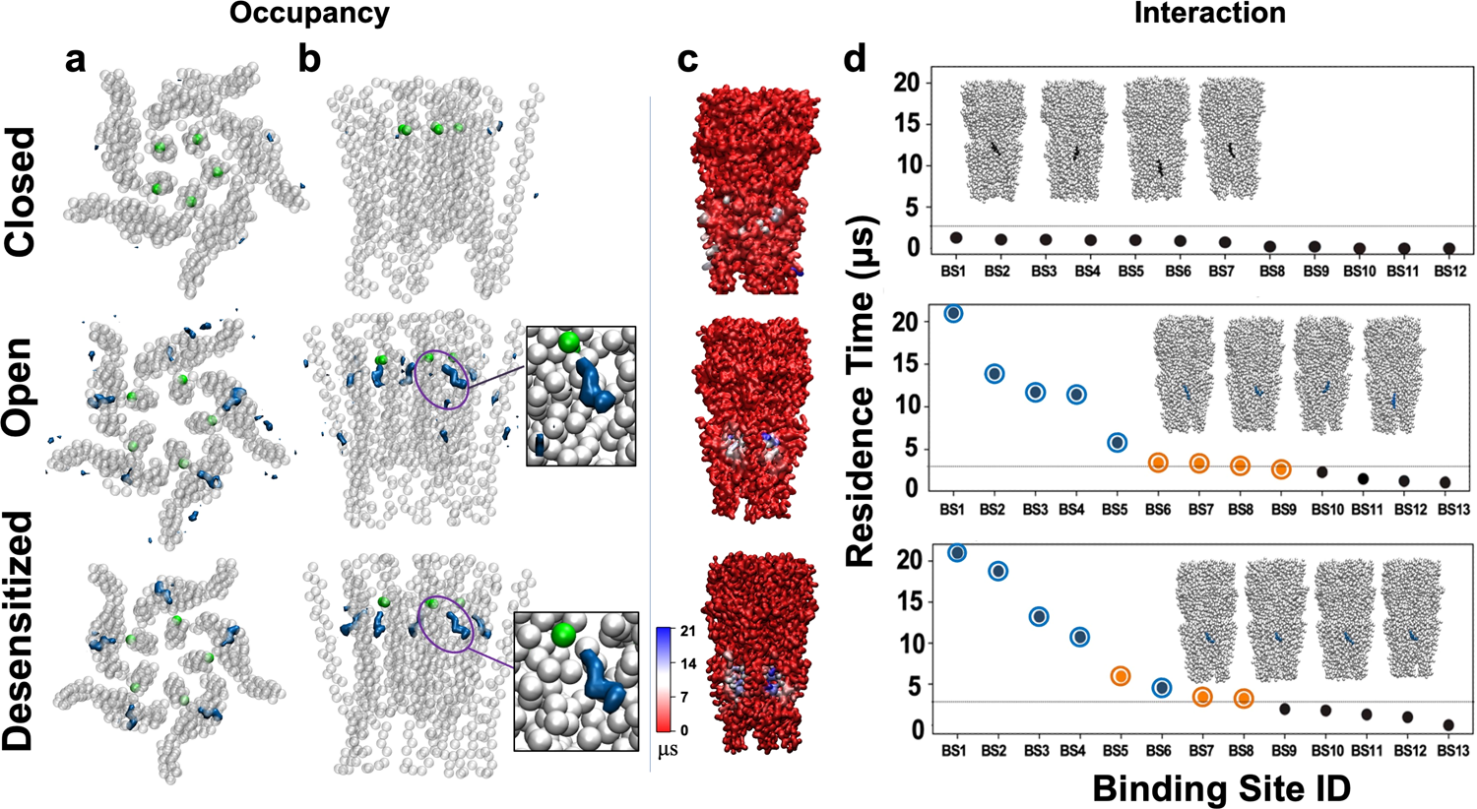
State-dependent site for cholesterol identified by coarse-grained simulations. (a) Densities (blue) representing >50% cholesterol occupancy around the GlyR TMD (gray), including the bead representing Ser-283 (green), viewed from the extracellular side. (b) Densities as in (a), viewed from the membrane plane. Insets show zoomed-in views of the high-occupancy region in the open and desensitized states. (c) Interaction residence times of cholesterol with protein residues calculated by PyLipID, shown as a molecular surface colored according to the scale at the bottom left (red–blue, 0–21 μs). (d) Interaction residence times of cholesterol at the top 12 individual PyLipID-identified binding poses. Poses with the four longest residence times (BS1–BS4) are illustrated above each plot, showing representative conformations of the protein (gray) and cholesterol (blue). Interaction times are shown for the five symmetrical intersubunit sites (cyan) as well as alternative sites of more (brown) or less (gray) than 3 μs (threshold line). Each column panel shows data for the closed (PDB ID 6PM3, top), open (PDB ID 6PM2, middle), and desensitized (PDB ID 6PM1, bottom) states.

In order to simulate interactions with physiologically relevant lipids, we first embedded each structure in an asymmetric bilayer designed to mimic a neuronal membrane^29,37^ (Figure 1) and ran four replicate 22 μs simulations of each structure. Filtering at 50% occupancy revealed no strongly preferred sites, including POPC, POPE, DOPS, SM, or PIP_2_. Conversely, a groove buried in the membrane outer leaflet between the M2 and M3 helices of one subunit and the M1 helix of the complementary subunit was distinctively occupied by cholesterol in simulations of both the open and desensitized states (Figure 3a,b). This interaction appeared to be state-dependent; in the closed state, cholesterol was observed to have a weak occupancy only around M4.

To verify this apparent state-dependent cholesterol site and quantify its dynamics, we used the PyLipID tool^47^ to identify discrete sites associated with the longest protein interaction residence times across all coarse-grained simulations of each system. Distinctive interaction hotspots for cholesterol were identified in both open and desensitized structures at the outer intersubunit cleft (Figures 3c and S2), corresponding to the regions of high occupancy described above. Indeed, in both states, the longest-lived cholesterol interaction poses — each with at least 3 μs residence time overlapped the high-occupancy site in at least four of the five equivalent subunit interfaces for all simulations (Figure 3d). Conversely, cholesterol interactions in the closed state were far shorter lived and broadly distributed.

### Cholesterol Stability and Interactions Revealed by Atomistic Simulations

**Figure 4:**
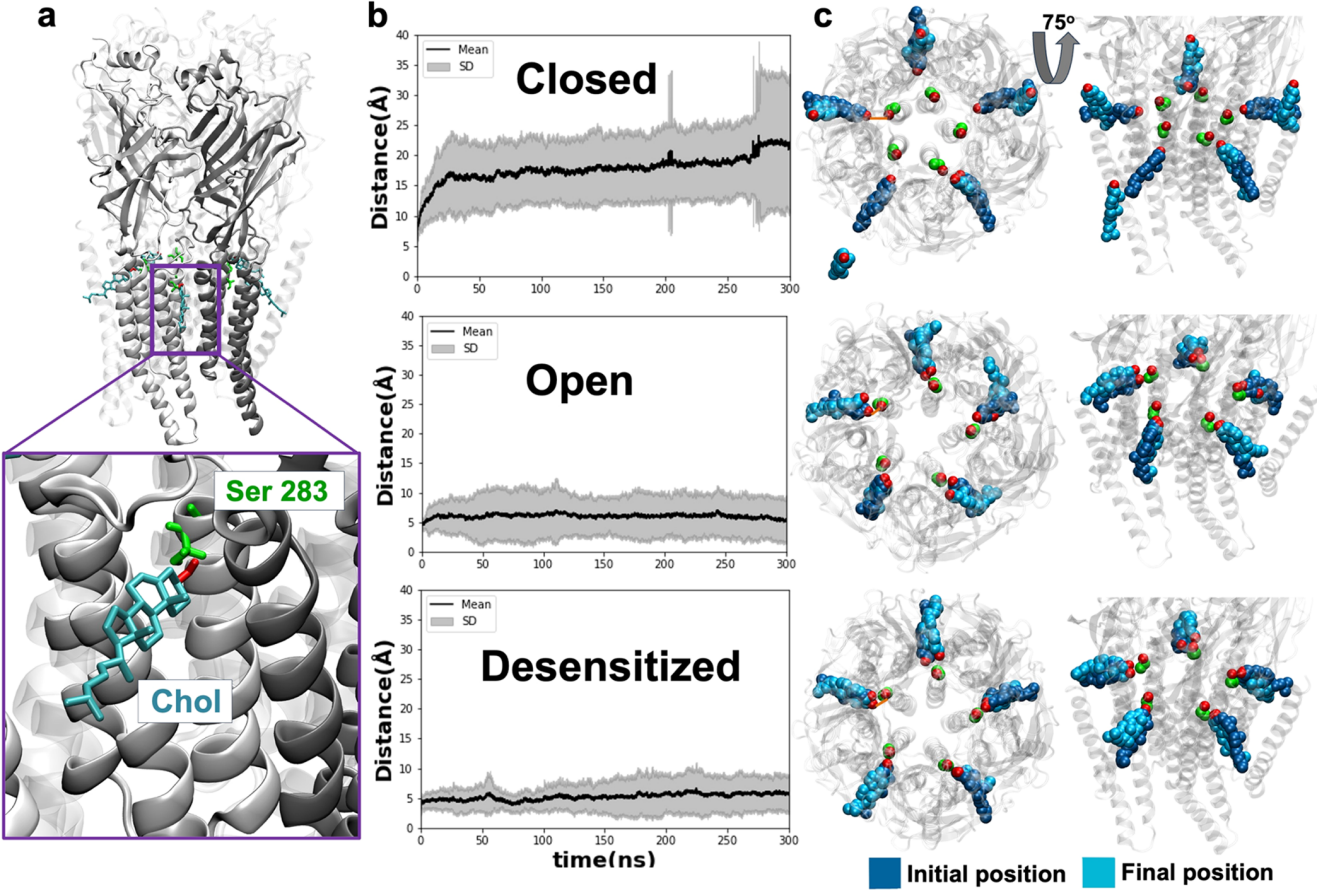
Cholesterol stability and interactions revealed by atomistic simulations. (a) Representative all-atom starting model of the GlyR (gray) with cholesterol molecules (Chol, cyan) bound at the intersubunit sites, proximal to Ser-283 (green). The inset shows a zoomed-in view of a single intersubunit site. For clarity, the principal and complementary subunits in the zoom-view site are shown as light and dark opaque ribbons, with the remaining subunit semitransparent. Cholesterol and Ser-283 are shown as licorice, colored by a heteroatom. **(b)** Mean distance (±standard deviation, SD, gray) between Ser-283 and its adjacent cholesterol molecule for all five subunits in all four replicas in each state. **(c)** Initial (1 ns, blue) and final (300 ns, cyan) positions of cholesterol molecules, colored by heteroatoms, in representative atomistic simulations of GlyRs (gray) in three states. Left-hand views show each system from the extracellular side; right-hand views show that from the membrane plane.

Having identified a putative cholesterol binding site specific to open and desensitized states of the receptor, we next sought to characterize amino acid contacts with cholesterol in detail using atomistic simulations in the smaller POPC bilayer (see the Methods). In the initial cholesterol pose, the hydroxyl moiety of cholesterol was oriented toward Ser-283 on the M2 helix of each subunit, with its multiring system bridging the subunit cleft and its hydrophobic tail projecting toward the lipid bilayer (Figure 4a). During the quadruplicate 300 ns simulations, cholesterol remained relatively close to Ser-283 in simulations of both the open and desensitized states (Figure 4b,c). Conversely, cholesterol was rapidly displaced from this initial orientation in closed-state simulations, often flipping, reorienting, or dissociating entirely from the receptor. A detailed description of further cholesterol contacts is provided below.

**Figure 5:**
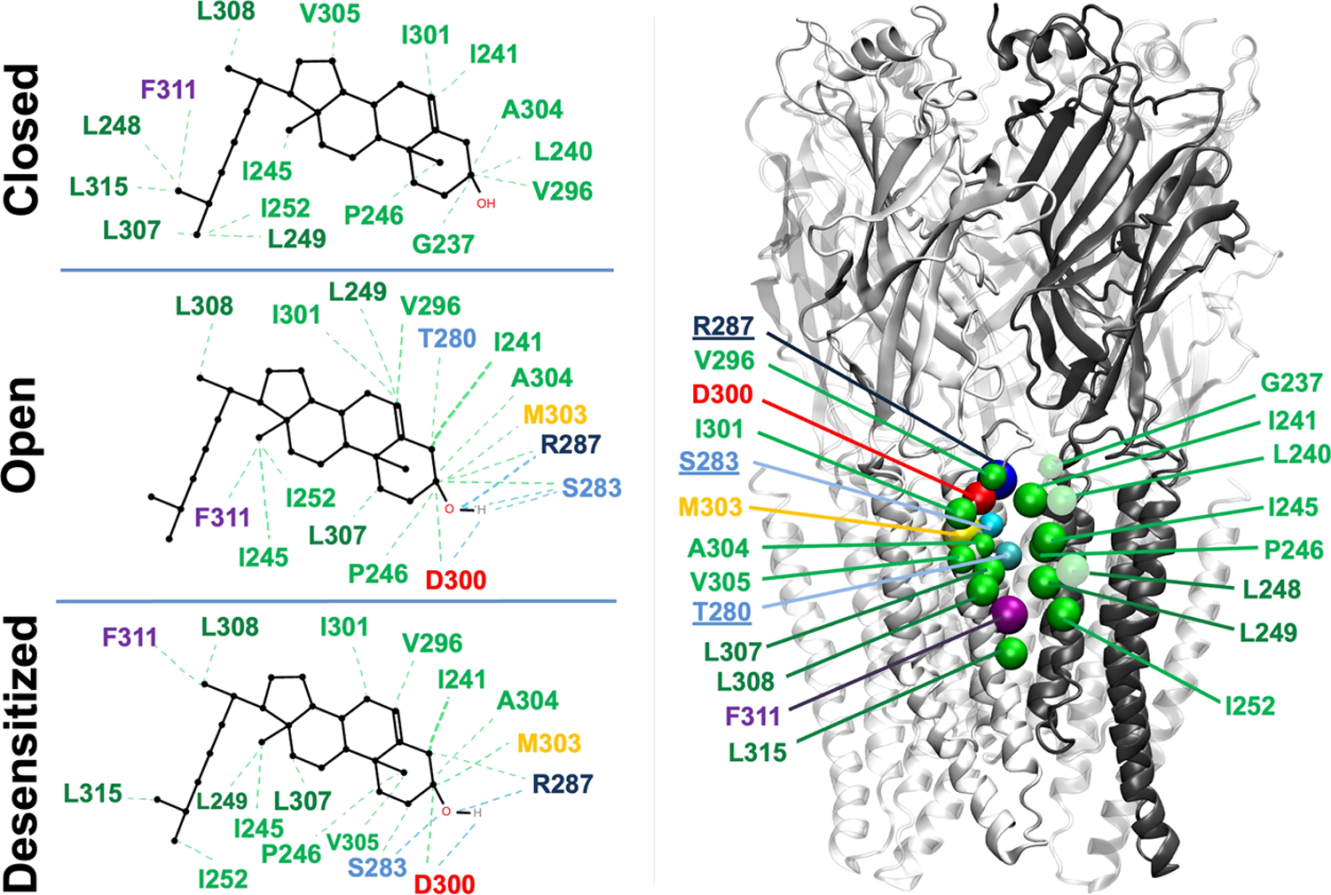
Cholesterol contacts the outer-leaflet interfacial site. Left-hand panels show interaction fingerprints for cholesterol generated in ProLIF from quadruplicate all-atom MD simulations of closed (top), open (middle), and desensitized (bottom) states of the receptor. The right-hand panel shows residues highlighted by ProLIF as Cα spheres on a representative GlyR model, depicted as in Figure 4a. Underlined residues are located on M2. The open and desensitized states share similar patterns, whereas the closed state displays weaker interactions and lacks hydrogen bond M2 contacts. In all panels, residues are colored by chemical property, including hydrophobic (green), aromatic (purple), acidic (red), polar (cyan), basic (navy), and sulfur-containing (yellow) side chains.

Amino acid interaction fingerprints generated in ProLIF^56^ identified a discrete cholesterol pocket surrounded by the M2 and M3 helices of one subunit and M1 of the complementary subunit in both open and desensitized states (Figure 5). In particular, residues Ser-283, Arg-287, and Asp-300 were capable of polar interactions with the cholesterol hydroxyl group, while bands of neighboring residues including Ile-241, Ile-245, Pro-246, Leu-249, and Ile-252 on M1; Thr-280 on M2; and Val-296, Ile-301, Met-303, Ala-304, Val-305, Leu-307, Leu308, Phe-311, and Leu-315 on M3 made frequent hydrophobic contacts with the steroid.

### Cholesterol-Binding Region Implicated in Channel Gating by Protein Perturbation Analysis

**Figure 6:**
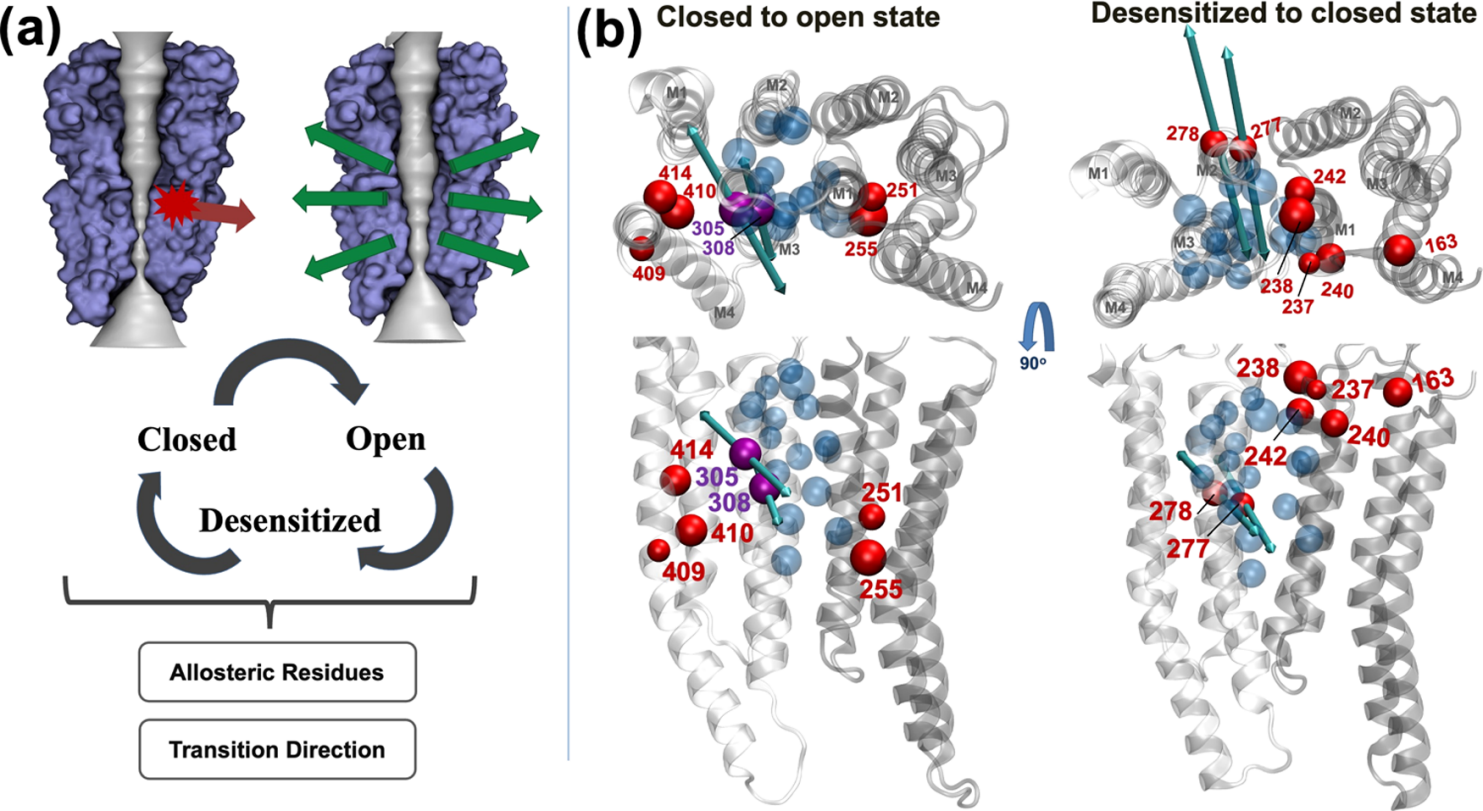
Cholesterol-binding region implicated in channel gating by protein perturbation analysis. (a) Schematic of protein perturbation calculations, in which a perturbation (red arrow) induces motions of residues (green arrows) that are assessed by their overlap with the direction of a relevant conformational change, in this case between closed, open, and desensitized states. (b) Single TMD subunit interface of the a1 GlyR is shown (gray ribbons), with key residues implicated in channel opening (closed-to-open transition, left) and in recovery from desensitization (desensitized-to-closed transition, right) shown as Cα spheres (red). Views are from the extracellular side (top) and the membrane plane (bottom). Residues Val-305 and Leu-308 (purple) were identified as both key residues in channel opening and as cholesterol contacts. Force vectors corresponding to the two residues with the highest overlap are shown as bidirectional arrows, colored cyan. For comparison, residues contacting cholesterol in atomistic simulations (as identified in Figure 5) are shown as transparent blue Cα spheres in VMD bead representation.

Since coarse-grained simulations typically require scaffold restraints on the protein structure and atomistic simulations struggle to cover time scales relevant for gating, the complete allosteric transitions underlying GlyR function are not easily modeled. We therefore employed a perturbation response scanning approach to identify residues whose motions correlate with conformational cycling.^33,34^ To initiate this analysis, we performed additional brief unliganded atomistic simulations in which the receptor did not deviate substantially from its initial structure (within 3.5 Å Cα-RMSD) or alter its pore conformation. Then, we generated the responses of all residues in the receptor to a perturbation inserted at a selected site (Figure 6). These perturbations mimic external forces such as random Brownian kicks or ligand binding. The objective is to find so-called allosteric residues whose displacements overlap most with the conformational change between the two states of a protein. This approach has been used to locate hotspots that influence protein dynamics in multiple systems.^33,34,57^ For the GlyR, we identified distinctive residue sets with relatively high overlap scores (>0.6) for the opening (closed-to-open) and recovery (desensitized-to-closed) transitions (Figure 6, Figure S1, Table 3), as detailed below.

**Table 3:** Summary of Protein Perturbation Results Including the Highest Overlap Values and Key Residues

protein perturbation	initial structure ID (simulation frame)	target structure ID	converged trajectory interval	average Cα-RMSD in interval	best overlap (*Oi*)	key residues
closed-to-open	6PM3 (180 ns)	6PM2	180–300 ns	2.8 Å	0.65–0.60	305, 308, 414, 255, 251, 409, 410, 306
open-to-desensitized^a^	6PM2 (60 ns)	6PM1	60–300 ns	3.1 Å	0.43–0.40	117, 171, 170, 229, 205, 167
desensitized-to-closed	6PM1 (150 ns)	6PM3	150–300 ns	2.8 Å	0.72–0.70	277, 278, 242, 237, 238, 163, 240, 267, 244

a The transition between open and desensitized states was not associated with any key residues with overlap >0.40, consistent with this being a relatively subtle/localized conformational change.

For the closed-to-open transition, perturbation analysis identified M1 residues Val-251 and Trp-255, M3 residues Val-305, Cys-306, and Leu-308, and M4 residues Ala-409, Phe-410, and Phe-414 as potentially allosterically relevant, with an overlap of 0.60–0.65 (Table 3, Figure S3). Notably, Val-305 and Leu-308 also frequented direct contacts with cholesterol in atomistic simulations, while Val-251, Trp-255, and Phe-414 occupied a proximal shell around the cholesterol site (Figure 6). For the recovery of the desensitized-to-closed state transition, relevant residues identified by perturbation analysis included Met-163 in the extracellular Cys loop, Gly-237, Tyr-238, Leu-240, Gln-242, and Tyr-244 in M1 and Ala-267, Leu-277, and Thr-278 in M2, with an overlap of 0.70–0.72 (Table 3, Figure S3). Again, several of these residues clustered around the proposed state-dependent cholesterol site (Figure 6). Force vectors associated with the highest-overlap residues relevant to both opening and recovery transitions were near-perpendicular to the pore axis, implying a blooming transition at the transmembrane interface (Figure 6). Thus, residues in or near the proposed state-dependent cholesterol site were implicated in transitions both from and toward the closed state.

### Differential Cholesterol Binding in Heteromeric GlyRs

**Figure 7:**
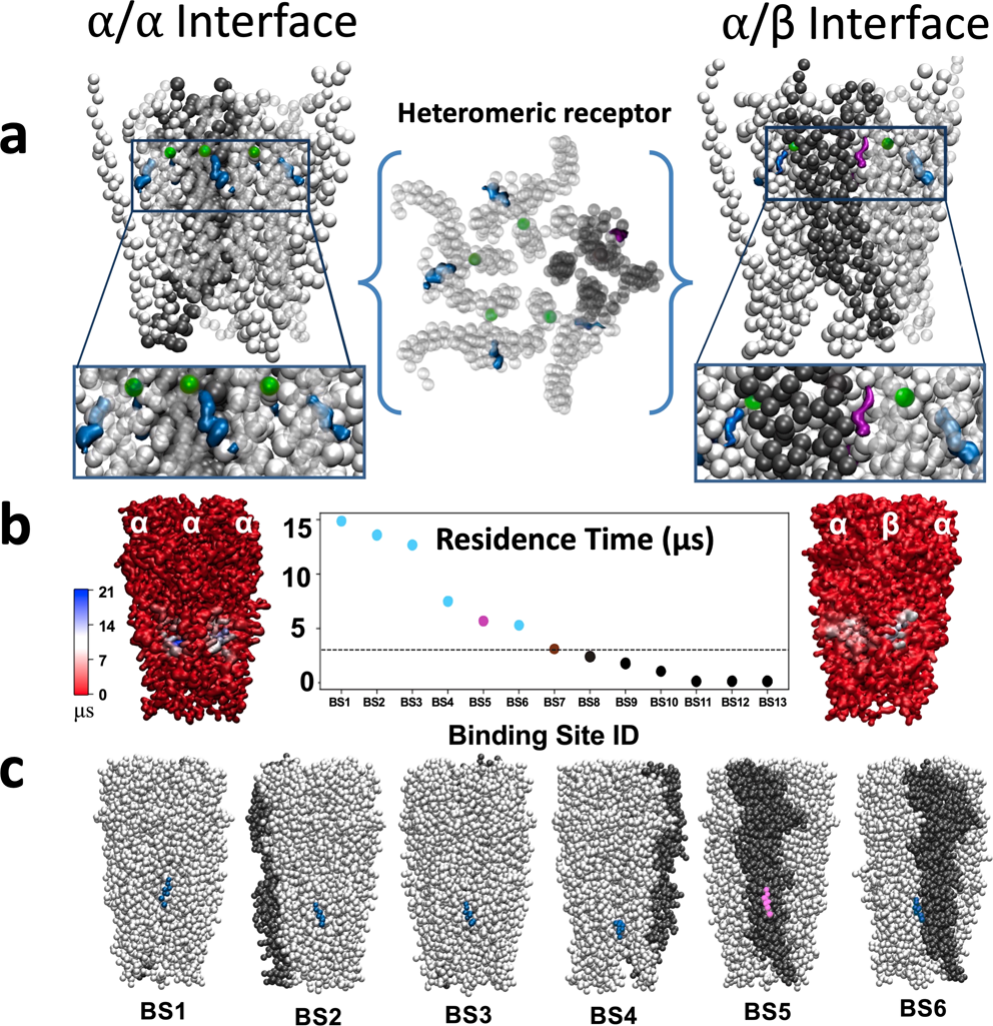
Differential cholesterol binding in heteromeric GlyRs. (a) Densities representing >50% cholesterol occupancy around α1 (blue) and β (purple) subunits of a heteromeric GlyR (gray), including a sphere representing Ser-283 (green). The center view is from the extracellular side; left- and right-hand views are from the membrane plane, focusing on different interface types. Insets show zoom views of the outer-leaflet interfacial site. **(b)** Interaction residence times of cholesterol at the top 13 PyLipID binding poses (center), with molecular surfaces focusing on different interfaces at left and right, colored as in Figure 3c. Interaction times are shown for intersubunit sites near the principal face of α1 (blue) or β (purple) subunits as well as alternative sites of more (brown) or less (gray) than 3 μs (threshold line). Poses with the six longest residence times (BS1–BS6) are illustrated below, showing representative states of the protein (gray) and cholesterol near the principal face of α1 (blue) or β (purple) subunits. One α–α interface is populated by two discrete cholesterol poses (BS1, BS3).

To test the relevance of our proposed cholesterol interactions in a potentially more physiologically relevant native heteromeric GlyR, we also ran coarse-grained simulations on the pig α1β structure recently reported in a desensitized state (PDB ID 7MLY).^13^ In this system, cholesterol molecules frequently occupied outer-leaflet sites at α–α and α–β interfaces (Figure 7a) overlapping those observed in homomeric receptors proximal to α1 Ser-283. Cholesterol interactions at these sites were also among the longest in duration (Figure 7b). Interestingly, cholesterol exhibited differential behavior at the β–α interface, where the residue equivalent to Ser-283 is cysteine. In this region, a site with cholesterol present for more than 3 μs duration (BS5) was still observed but located superficially on the surface of the β subunit rather than buried at the subunit interface.

## Discussion and Conclusions

Allosteric gating and modulation are critical to the function and pharmacology of many membrane proteins, including pLGICs. Although 3D structures are increasingly available for this receptor family, intrinsically dynamic allosteric processes may not be clearly described by static structural data, in particular, not in the context of interactions with other molecules such as lipids. By combining coarse-grained and atomistic simulations as well as perturbation-based computational analyses of three structures determined under identical experimental conditions, we defined molecular details and mechanistic relevance of a state-dependent cholesterol-binding site in a zebrafish GlyR. Additionally, we report that the pig heteromeric GlyR, with 99% identity to the human protein sequence, contains a similar putative binding site at all subunit interfaces except for β–α.

**Figure 8:**
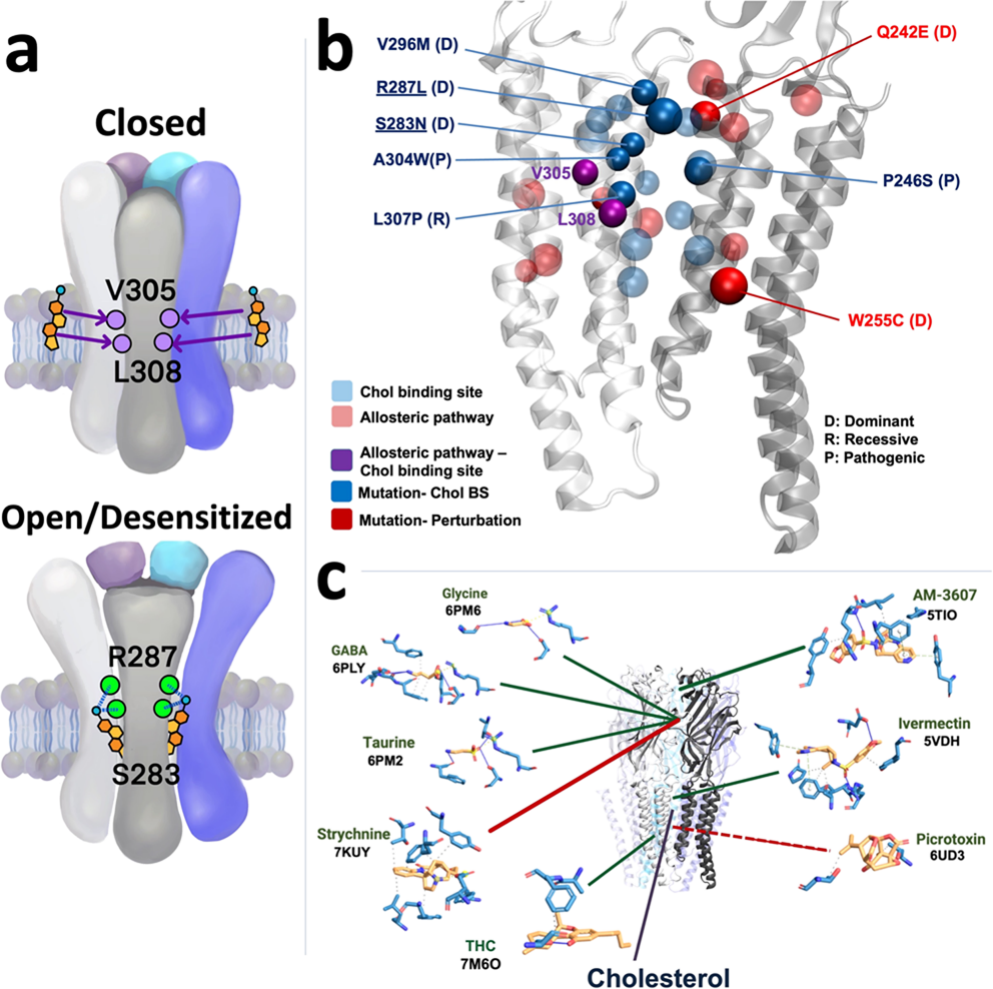
Functionally important GlyR residues implicated in cholesterol binding and/or allostery. (a) Proposed state-dependent site for cholesterol, with a preference for binding in the open and desensitized states over the closed state. The top model indicates the potential involvement of residues such as Val-305 and Leu-308 (purple) in binding cholesterol (based on atomistic simulations) and driving channel opening (based on perturbation analysis). The bottom model indicates potential stabilizing contacts of cholesterol in the open and desensitized states with the polar residues Ser-283 and Arg-287 (green) on the pore-lining M2 helix. (b) Solid spheres indicate correspondence between previous evidence for functional relevance and residues implicated here in cholesterol binding (blue), allosteric transitions (red), or both (purple). Residues linked to hyperekplexia are labeled as dominant (D), recessive (R), or indeterminately pathogenic (P). The right-hand labels are associated with M1 of the complementary face, while the left-hand labels correspond to M2 and M3 of the principal face; underlined residues are located in M2. (c) Ligands (gold) resolved in past experimental GlyR structures with directly coordinating residues (blue). For clarity, the two proximal subunits are shown in gray and black; the remaining are semitransparent.

In our simulations, cholesterol was capable of intercalating into the outer transmembrane subunit cleft, deep enough to interact directly with residues from the principal-face pore-lining M2 helix. The residue Ser-283 (15′, Ser-267 in humans) emerged as a particularly informative location for state-dependent binding, often capable of forming a hydrogen bond with the cholesterol hydroxyl group in simulations of open or desensitized receptors. Differential orientation of this residue in the closed state appeared to preclude cholesterol interactions, reducing cholesterol occupancy at the interfacial site. Sequence variation at this position in GlyR β subunits was further associated with more superficial interactions with this component of a heteromeric receptor. Mutations at Ser-283 have long been shown to alter glycine sensitivity^60^ and are linked to autosomal dominant hereditary hyperekplexia, a rare but potentially lethal neuromotor disorder caused by abnormal glycinergic transmission^61^ (Figure 8a, b). Cholesterol also made frequent polar contacts with the M2 residue Arg-287 (19′, Arg-271 in human), one turning outward from Ser-283 at the extracellular mouth of the pore. This residue has been termed as a “gating mutation,” as mutations appear to uncouple agonist binding from gating, reduce agonist efficacy, and even convert partial agonists into antagonists. Arg-287 is also one of the most common sites of hyperekplexia mutations^61,62^ (Figure 8a, b). Interestingly, the equivalent position has also been linked to pathology in nAChRs, where cholesterol has been shown to bind the outer-leaflet site in a state-dependent manner.^63,64^

Extending from the channel pore toward the membrane, several residues making hydrophobic contacts with cholesterol in simulations have also been implicated in channel function (Figure 8a). On the principal M3 helix, substitutions at Val-296 (to Met) and Ala-304 (to Trp) produce tonically open leaky channels, while those at Leu-307 alter glycine sensitivity, surface expression, and/or desensitization.^65−67^ Mutations at Val-296 and Leu-307 are also associated with hyperekplexia.^68,69^ On the complementary M1 helix, a hyperekplexia-linked Ser substitution at Pro-246 decreases glycine sensitivity and surface expression.^65^ The substitution P246S, heterozygous with R81W, also produces fast-desensitizing receptors.^61,65^ Similarly, substituting a bulky Trp one turn inward at Leu-249 enhances desensitization in the presence of ivermectin.^66^ Thus, although functional evidence for cholesterol contacts in GlyRs is limited, specific state-dependent interactions identified in this work are consistent with an influential region of channel function. Peripheral to these direct cholesterol contacts, several of the residues (Gln-242, Tyr-244, Trp-255, Ala-267) we identify (Table 3) as relevant for allosteric gating have also been linked to hyperekplexia^61,65,69−72^ (Figure 8b). Substituting Glu at the M1 residue Gln-242 produces leaky channels, possibly due to an enhanced interaction with the M2 residue Arg-287 described above.^65,69^ Conversely, substituting Glu at the M2 residue Ala-267 in the inner mouth of the pore suppresses recovery from desensitization.^72^ These effects support the utility of perturbation analysis in identifying amino acid residues critical to channel function independent of cholesterol.

The outer transmembrane site we associated with cholesterol may overlap sites for other modulators (Figure 8c). In particular, structures of GlyR bound to the lipophilic potentiator ivermectin reveal direct interactions with Ile-241, Gln-242, Pro-246, Ser-283, and Ala-304, all frequent contacts of cholesterol. Indeed, residues Pro-246 and Ala-304 were shown to be crucial determinants of ivermectin sensitivity in a TMD mutagenesis screen; the extreme substitution A304F eliminates ivermectin activity altogether.^66,73^ Smaller drugs such as alcohols, anesthetics, cannabidiol, and quercetin have also been associated with the same region in various pLGICs, including influential effects of mutations at Thr-280,^67,74^ Ser-283,^3^ Met-303,^74^ and Ala-304.^73,75^ Dicysteine cross-linking studies show that Ala-304 is proximal to both Ser-283 and Ile-245, with double mutations producing leaky channels which function normally after reduction of the disulfide bond.^75^ These functional results support a role for the outward-facing subunit interface in GlyR gating and drug modulation, particularly residues on M2 and M3 that are also implicated here in cholesterol binding.^76^

Tetrahydrocannabinol (THC) was also recently observed in a GlyR transmembrane site in contact with the residue Phe-410,^77^ which was implicated here in GlyR opening. Interestingly, cholesterol depletion has been shown to mimic disruption of THC binding.^3,78^ It is plausible that this drug modulates channel function in part by piggybacking a site evolved for state-dependent binding of endogenous cholesterol. Recent coarse-grained simulations (in the absence of cholesterol) support a similar intrasubunit site for THC binding and indicate that the endogenous cannabinoid N-arachidonyl-ethanolamide (AEA) binds in both this THC site and the intersubunit ivermectin site.^75^

Our simulations offer testable hypotheses for cholesterol modulation of GlyRs, an effect for which evidence has been limited. In contrast to proteins such as dopamine transporters, GPCRs, and sodium–potassium pumps^79−81^ with which cholesterol has been shown to crystallize, cholesterol binding to GlyRs has yet to be definitively observed in experiments. Lipidic densities in GlyR structures have generally been modeled as partial phospholipids.^11,82^ Interestingly, the intersubunit site identified in our work does not contain the proposed cholesterol-sensing CRAC or CARC motifs.^83,84^ Experiments using mass spectrometry identified cholesterol contacts in the pre-M1, M2–M3 loop, intracellular, and M4 regions but not in the intersubunit cleft identified here; given that the relevant protocol was likely limited to closed receptors, it is plausible that alternative interactions in that state poise cholesterol for entering the intersubunit pocket upon activation, although simulations indicated that such sites are only weakly occupied, even in closed channels.^85^

Our findings build on those from previous coarse-grained simulations, particularly from homology models of closed and open human GlyRs.^29^ While that work offered a detailed methodological pipeline and characterized interactions with multiple lipids, here, we proceeded from coarse-grained observations to detailed amino acid contacts and dynamics of allostery using atomistic simulations and perturbation analysis. Moreover, since the initiation of that work, several new GlyR experimental structures have emerged, in some cases casting doubt on the physiological relevance of homology-modeling templates. Our present work is based on three structures determined under identical conditions, allowing insights into the desensitized state as well as a potentially more relevant open state. In future studies, it may be particularly interesting to probe functional consequences of residues such as Val-305 and Leu-308, implicated here both in binding cholesterol and driving channel opening (Figure 8a).

These findings could further support the discovery of new ligands that can modify or substitute for cholesterol interactions. Various hydrophobic compounds including coenzyme Q10, α-tocopherol, and vitamins D3 and K1 can attach to cholesterol sites.^17^ In particular, GlyRs are modulated by several steroid hormones, which are structurally similar to cholesterol and function through shared binding sites. Stress hormones including corticosterone, cortisol, and their metabolites have been shown to promote GlyR desensitization in neurons, potentially linking to brain dysfunction in chronic stress.^86^ Moreover, modulation of GlyRs by neurosteroids appears to be subunit-dependent: the presence of β-subunits reduces their sensitivity, possibly due to a decrease in available binding sites^87^ as indicated here for cholesterol.

## Data Availability

Simulation frames and parameters for both coarse-grained and atomistic simulations can be accessed via the DOI: 10.5281/zenodo.8374103. The simulation setup and execution are explained in the Introduction to Membrane-Protein Simulation GROMACS tutorial at the DOI: 10.5281/zenodo.10952993.

## Supporting Information Available

Structural stability of GlyR models evaluated through RMSD analysis and pore characterization, PyLipID analysis revealing nine distinct cholesterol interaction poses across the two states, and potentially allosterically relevant residues (PDF)
